# A Novel On-Chip Liquid-Metal-Enabled Microvalve

**DOI:** 10.3390/mi12091051

**Published:** 2021-08-30

**Authors:** Jiahao Gong, Qifu Wang, Bingxin Liu, Huimin Zhang, Lin Gui

**Affiliations:** 1 Liquid Metal and Cryogenic Biomedical Research Center, Technical Institute of Physics and Chemistry, Chinese Academy of Sciences, 29 Zhongguancun East Road, Haidian District, Beijing 100019, China; gongjiahao18@mails.ucas.ac.cn (J.G.); liubingxin17@mails.ucas.ac.cn (B.L.); zhanghuimin19@mails.ucas.ac.cn (H.Z.); 2 School of Future Technology, University of Chinese Academy of Sciences, 19 Yuquan Road, Shijingshan District, Beijing 100039, China; 3 Department of Mechanical Engineering, Villanova University, 800 Lancaster Avenue, Villanova, PA 19085, USA; qwang5@villanova.edu

**Keywords:** liquid metal microvalve, repeatability, easy fabrication, high switching ratio

## Abstract

A room temperature liquid metal-based microvalve has been proposed in this work. The microvalve has the advantages of easy fabrication, high flexibility, and a low leak rate. By designing a posts array in the channel, the liquid metal can be controlled to form a deformable valve boss and block the flow path. Besides, through adjustment of the pressure applied to the liquid metal, the microvalve can perform reliable switching commands. To eliminate the problem that liquid metal is easily oxidized, which causes the microvalve to have poor repeatability, a method of electrochemical cathodic protection has been proposed, which significantly increases the number of open/close switch cycles up to 145. In addition, this microvalve overcomes the shortcomings of the traditional microvalve that requires an alignment process to assemble all the parts. When the valve is closed, no leak rate is detected at ≤320 mbar, and the leak rate is ≤0.043 μL/min at 330 mbar, which indicates it has good tightness. As an application, we also fabricate a chip that can control bubble flow based on this microvalve. Therefore, this microvalve has great prospects in the field of microfluidics.

## 1. Introduction

Microvalves are essential components in the field of microfluidics [1,2,3,4]. There are many applications, such as fluid mixing [5,6], droplet fusion [7], nucleic acid manipulation [8,9], and cell culture [10,11]. Microvalves can also change the resistance of the fluid in the channel and the shape of the flow channel with the help of a micro-pump. The presence of microvalves greatly reduces manual labor and increases the efficiency of microfluidic control [12].

The materials of microfluidic chips are typically polydimethylsiloxane (PDMS). There are many studies on all kinds of PDMS-based microvalves, including electrostatic [13,14], electrokinetic [15], piezoelectric [16], phase change [17], electromagnetic [18], and SMA [19] studies. Among them, the most common and simplest structure is the Quake’s valve [20]. This valve has three-layer architecture made in PDMS: a microchannel lying orthogonally on top of another microchannel with a thin elastomeric membrane between them. One of the microchannels acts as a “control channel” that, when pressurized, causes deflection of the membrane to interrupt the flow in the orthogonal “flow channel”. However, a multi-layer structure needs high-precision alignment technology for valve fabrication and location. Special treatment is also required to make the microchannel round instead of rectangular to minimize the dead volume of the Quake’s valve. Gui et al. proposed an SMA valve that can achieve high performance in a flow channel with a large aspect ratio [21]. Lee et al. proposed using 3D printing technology to make non-rectangular channels. This structure improved the switching performance of the valve to some extent [22]. However, these valves also often require complex multi-layer structure design and high-precision alignment.

Low-melting-point metals or alloys (gallium, gallium-based alloys, and bismuth-based alloys), as “injectable metals”, have been recently introduced and widely developed in the microfluidic field. Liquid metals have been used in almost all basic aspects of microfluidic systems—such as electrodes [23], electroosmotic pumps [24], sensors [25,26], and antennas [27]—except microvalves. Gao et al. successfully integrated liquid-metal-based microfluidic elements into one microfluidic system to fabricate a fast thermal response microfluidic system, including liquid metal micro-heaters, liquid metal temperature sensors, and liquid metal electroosmotic flow pumps [28]. This microfluidic chip is totally soft because it has only two kinds of soft materials inside, which are PDMS and liquid metal. The soft chip has potential applications many areas, such as wearable devices. However, this system had no valves. Therefore, this kind of system lacked an essential basic element, the “liquid metal-based microvalve”, which is responsible for controlling the on/off switch of fluid channels and fluid flow direction, so the fluid could not arrive at the specified sensor area within the specified time. There are also some studies on liquid-metal-based valves. Debray et al. manufactured disposable valves with low leakage by using low-melting alloys [29], which relied on mechanical strength changes caused by changes of alloy state through temperature regulation to achieve the valve function. It worked as a one-shot microvalve, but it was not soft enough due to the solid alloy at room temperature and the chromium–copper multilayer in the structure. Shaikh et al. utilized the properties of solidification and melting of low-melting alloys to lock and open the valves [30]. However, solid–liquid phase change increased the response time of the valve unexpectedly, and a multi-layer alignment process was required. It also had other issues, such as membrane collapse and stillation. Pekas et al. proposed using electrostatic forces between liquid metal and ITO to achieve valves’ on/off switching [31]. However, this valve also required an alignment process, and ITO is of poor flexibility and increases the chip cost.

In this work, a room temperature liquid metal-based microvalve with simple fabrication has been proposed. First, this microvalve avoids the complex alignment process of many other microvalves, such as the Quake’s valve [20], thus increasing the success rate of chip fabrication. Second, it is a total soft metal microvalve because it consists only of liquid metal and PDMS, and also, no phase transition is required compared with Shaikh’s valve [30]. Therefore, it is suitable for integration with wearable devices. The liquid metal we use in this work is GaInSn. GaInSn is nontoxic and has a low melting point of only 11 °C. Due to good flowability and high surface tension, GaInSn can be pumped into the specified flow channel without leakage and works like a “liquid metal tongue” with high tensile strength to block the flow. Third, due to less dead zone being produced, it can present excellent anti-leakage characteristics, with a leak rate ≤0.045 μL/min at 330 mbar, which proves that the valve has good tightness. In addition, the maximum burst pressure of 390 mbar also reflects the good shock-resistance of the valve. Because the liquid metal oxides stuck to the PDMS may affect the repeatability of the valve, electrochemical cathodic protection has been verified as an effective way to prevent oxidation [32] to improve the repeatability. Based on this work, we also fabricate a microfluidic chip that can control the direction of bubble flow as a possible application.

## 2. Experimental Details

### 2.1. Design of the Microvalve

**Figure 1:**
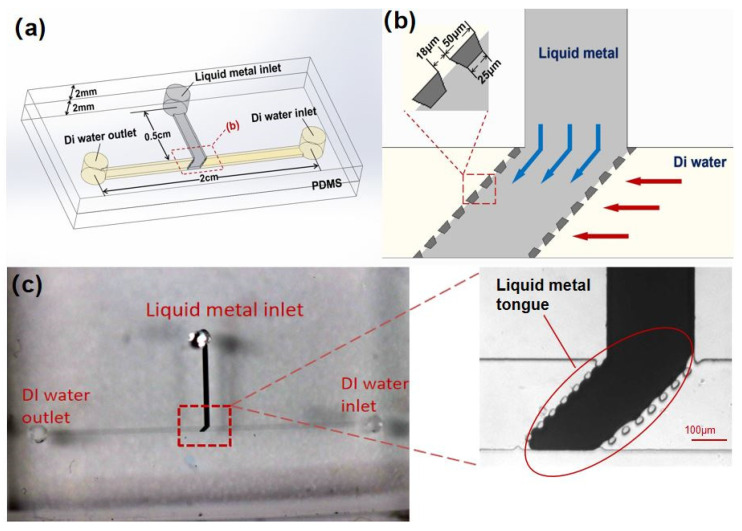
Structure diagrams of the microvalve. (**a**) Schematic of “T”-shaped microvalve; the vertical channel was filled with liquid metal, and lateral flow channel was filled with the sample fluid. (**b**) The plane structure of the microvalve with an inclination angle of 45°. (**c**) Photographs of the microvalve and its micro-structure under a microscope.

The liquid-metal-based microvalve chip was designed to control micro-fluidic motion. As shown in Figure 1a, this valve consisted of two layers: the upper layer was a PDMS slab (2 mm thick) embedded with a “T”-shaped microchannel (50 μm high). The lower layer was another PDMS slab (2 mm thick) as the base without any structures. The vertical channel (0.5 cm long, 300 μm wide) was a liquid metal flow path and the horizontal channel (2 cm long, 300 μm wide) was a fluid sample flow path. Two columns of PDMS posts were juxtaposed and equally spaced at the intersection of the liquid metal and the fluid sample flow path. Figure 1b shows the partially enlarged view of the PDMS posts. The shape of the post is isosceles trapezoidal with an upper base width of 25 μm, a lower base width of 40 μm, and a height of 25 μm. The minimum gap between the posts was 18 μm. These parallel post columns were the core structure in the valve which prevented liquid metal from entering the fluid sample channel. The posts were also designed at different inclination angles for parametric study, including 30°, 45°, 60°, 75°, and 90°.

### 2.2. Fabrication of the Microvalve

The liquid metal microvalve chip was fabricated by standard soft lithography. First, a SU-8 2050 (MicroChem, Westborough, MA, USA) was used to make the mold of the T-shaped channel with a height of 50 μm on silicon wafers (Ultrapak^®^ 100 mm, Entegris, Billerica, MA, USA). Second, a Sylgard 184 silicone elastomer (PDMS) (a mixture of a base and curing agent at a ratio of 10:1 by weight, Dow Corning, Midland, MI, USA) was used for casting on silicon wafers to form transfer patterns. After baking at 65 °C for 2.5 h, the cured PDMS can be peeled off from the silicon wafer. Then, the PDMS was cut into cuboid slabs (2.5 cm × 1.5 cm × 2 mm) that contain the entire channels. Third, another PDMS slab without any structures (2.5 cm × 1.5 cm × 2 mm) was used for plasma bonding with the previous channel layer (plasma cleaner, YZD08-2C, Tangshan Yanzhao Technology). After baking at 95 °C for 10 min, the fabrication of the microvalve chip was completed. In this work, the liquid metal Ga_66_In_20.5_Sn_13.5_ (weight percent, Ga 66%, In 20.5%, Sn 13.5%; melting point: 10.6 °C; ShanxiZhaofeng Gallium Co., Ltd., Quanyang, China) was prepared to work as the valve material, and DI water (Merck Chemical Technology CO., Ltd., Shanghai, China) was used as the fluid sample. Figure 1c shows the photograph of the microvalve and its microstructure under a microscope (Axio Observer Z1, Carl Zeiss, Jena, Germany). The liquid metal filled the whole vertical channel working as the valve, and the position between the posts is “liquid metal tongue”. The flow direction of DI water was from inlet to outlet.

### 2.3. Device Operation

**Figure 2:**
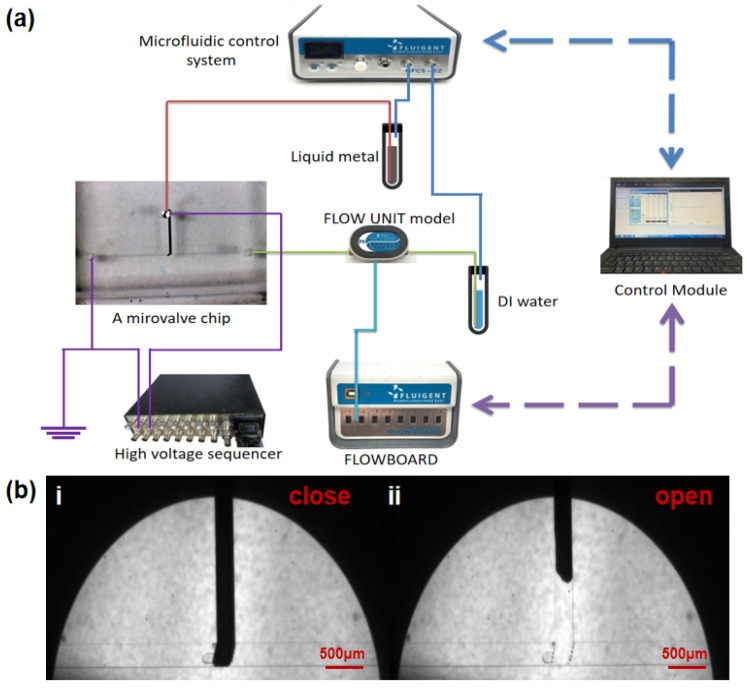
The control and flow detection platform of a microvalve and a valve switch diagram. (**a**) A computer works as a control module connected to a four-channel microfluidic control system (for pumping the liquid metal and Di water into the microchannels), and a flow rate platform (includes a FLOWBOARD and a FLOW UNIT model) to detect the flow rate of Di water. A high voltage sequencer is used to apply cathodic protection: the liquid metal inlet is connected to the cathode, and the anode is connected to the Di water outlet and grounded separately. (**b**) The close (**i**) and open (**ii**) state of the microvalve observed by a microscope.

The microvalve control and detection system is shown in Figure 2a. The switching of the microvalve is achieved by a four-channel microfluidic control system (MFCSTM-EZ, FLUIGENT, France) that regulates the pressure in the liquid metal flow path and the fluid sample flow path separately. Before pumping the liquid metal, the whole channel was filled with DI water to expel the air from the flow channel. The microvalve is opened at this moment. When the microvalve needs to be closed, the liquid metal is then pumped into the vertical channel. Due to the surface tension of the liquid metal, it can fill the entire vertical channel without flowing out of the gap between the PDMS posts to contaminate the sample flow path. At this time, the liquid metal completely block the fluid sample flow path, which prevents the sample from flowing through, thereby realizing the closing of the valve shown in Figure 2b(i). When the microvalve needs to be re-opened, the liquid metal pumping pressure is reduced, and the sample pumping pressure is increased at the same time. The pressure of the sample not only has a horizontal leftward push but also an upward push on the liquid metal. As a result, the sample pressure will gradually force the liquid metal to flow back to the vertical channel. Then the sample can flow normally from left to right, thereby realizing the opening of the valve shown in Figure 2b(ii).

To detect the switching performance of the microvalve, a Flow Rate Platform (MFCSTM-EZ, FLUIGENT, Villejuif, France) was used to monitor the change of the flow rate of the fluid sample. The minimum flow measured by the flow rate platform is 7 nL min**^−^**^1^, so it is sensitive to small leakage of the microvalve. By using this device, properties of the microvalve can be tested, including switching ratio, response time, and repetitiveness. It is noted that the microvalve chips need to be observed under a microscope during the whole process.

To increase the repeatability of the microvalve, a high-voltage sequencer (HVS448 6000D, LabSmith, Inc., Livermore, CA, USA) was used to apply negative voltage to the liquid metal to remove the oxide layer using the electrochemical protection method. As shown in Figure 2a, the inlet of the liquid metal microchannel is connected to the cathode. The anode is connected to the DI water and grounded separately. At this time, hydrogen is produced on the surface of the liquid metal by water electrolysis. Hydrogen plays the role of “mechanical stripping” on the surface of the electrode. Then, due to the high migration rate of hydrogen ions, there will be plenty of H+ aggregation near the cathode. The acidic environment on the electrode surface will also prevent the electrode from oxidation (Figure S1).

## 3. Results and Discussion

### 3.1. Valve Closing and Opening

**Figure 3:**
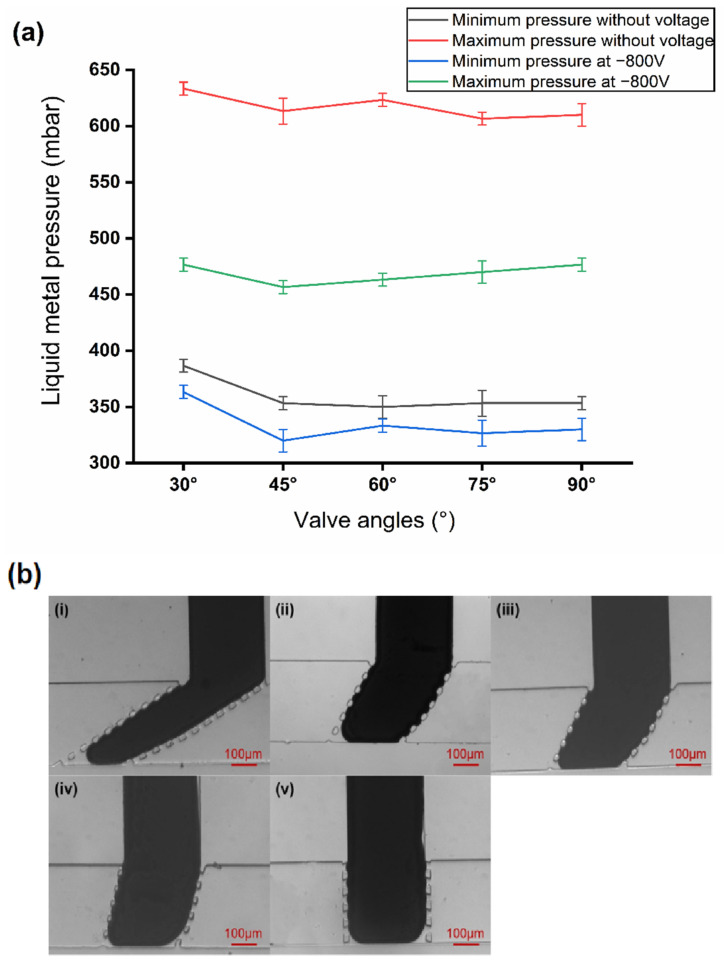
Study on valve closing conditions and valve closing diagram. (**a**) Liquid metal pressure ranges required for valve closing with and without voltage applied. (**b**) Different types of microvalves in closed state: (**i**) 30° inclination angle, (**ii**) 45° inclination angle, (**iii**) 60° inclination angle, (**iv**) 75° inclination angle, (**v**) 90° inclination angle.

To ensure that the valve can be closed and opened properly, electrochemical protection was required to remove the liquid metal oxides effectively in the switch operation. However, when a voltage (−800 V in this work) is applied on the liquid metal, electrowetting will occur, thus reducing its surface tension [33]. In this case, the liquid metal will rush out from the post gap more easily, with the maximum closing pressure reduced. Experiments were performed to study the range of pressure required to close the microvalve with and without −800 V applied. The test results are shown in Figure 3a. When −800 V is applied, the closing pressure of liquid metal for all angles is from 363.3 ± 5.8 mbar to 457.7 ± 5.8 mbar. When no voltage is applied, the closing pressure is from 386.7 ± 5.8 mbar to 606.7 ± 5.8 mbar. This indicates that the voltage applied reduces the closing pressure for liquid metal. Figure 3a shows the pressure range of the liquid metal required to close the valve for all the angles. The pressure range for −800 V and 0 V has an overlapped area between the green line and black line. Any pressure in this overlapped area can make sure the valve is properly closed, whether the −800 V is applied or not. Therefore, for convenience, we select a liquid metal pressure of 400 mbar in this area as a proper closing pressure for all the valves. During the experiments, when the 400 mbar is applied on the liquid metal, no leakage is detected when the pressure of fluid is lower than 330 mbar. Figure 3b shows the morphology of the microvalves with different inclination angles when they were fully closed at 400 mbar with −800 V applied.

When the valve needed to be opened, the pressure in the liquid metal flow path was quickly reduced from 400 mbar to 100 mbar, while the pressure in the sample flow path was set between 100 mbar and 200 mbar. Then, the liquid metal gradually returned to the inlet of the liquid metal channel under the combined action of the fluid pressure and the liquid metal tension, that is, the valve was in a fully open state, and then the fluid pumping pressure could be reduced to 0.

### 3.2. Electrochemical Protection Test

**Figure 4:**
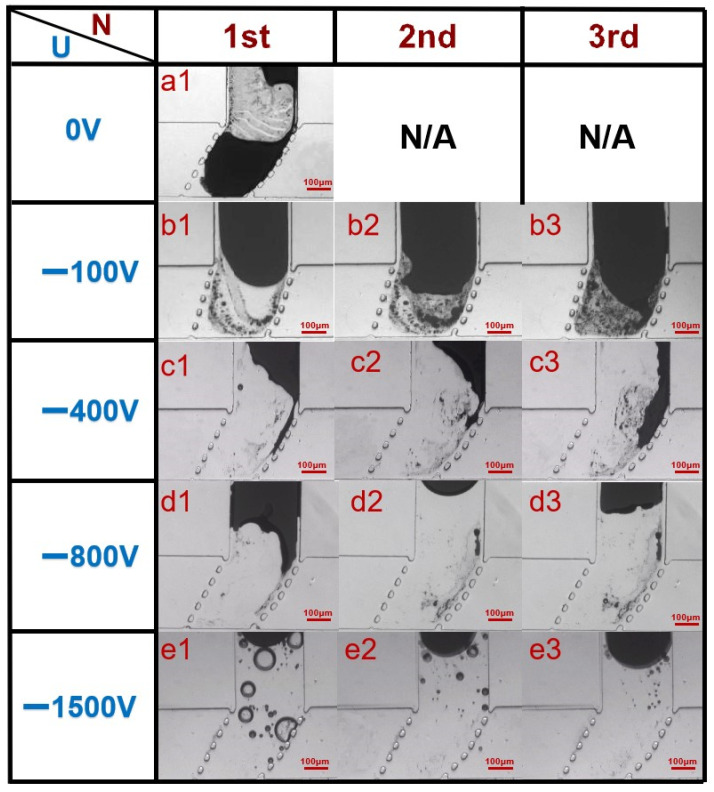
Diagram of the first three openings of the valve under different voltages: (**a1**) without cathodic protection; (**b1**–**b3**) opening status of the valve the first three instances at −100 V; (**c1**–**c3**) opening status of the valve in the first three instances at −400 V; (**d1**–**d3**) opening status of the valve in the first three instances at −800 V; (**e1**–**e3**) opening status of the valve in the first three instances at −1500 V.

To achieve normal on/off switching of the valve and improve its repeatability of use, electrochemical protection is adopted. The effect on oxide removal was discussed first to find out the optimal value for protective voltage. Thus, the effects of different voltages in electrochemical cathodic protection were studied. Figure 4a1 shows the operation of a liquid metal valve without cathodic protection. After opening the valve for just the first time, a large amount of metal oxide remained in the liquid metal flow channel; this is because the main composition of the oxide is Ga_2_O_3_, and Ga_2_O_3_ has strong wettability on the surface of many solid materials, including PDMS. The liquid metal could not be completely retracted to the vertical channel due to the adhesion of the metal oxide to the wall, which causes valve failure.

As control experiments, voltages of −100 V, −400 V, −800 V, −1500 V were respectively applied to the liquid metal in different microvalves. (The voltage is high because of the high electrical resistance of the DI water in microchannel.) Figure 4 shows the first three instances of valve opening at different voltages. At −100 V (Figure 4b1–b3), many oxides stuck to the wall, even when the valve was opened for the first time, and for the second and third time opening, accumulation of a large amount of oxide caused large areas of liquid metal to be unable to completely retreat from the posts. At −400 V (Figure 4c1–c3), the removal effect of oxide was obviously improved, and most of the liquid metal could retreat from the posts, but there were still residues remaining in the channel, especially on the right edge. During the third opening, too much liquid metal residue on the wall caused valve failure. At −800 V (Figure 4d1–d3), the removal effect of oxide was recognized as an ideal situation, and all three openings could be carried out successfully. It indicated that a higher voltage was more useful in eliminating oxide, showing the better performance of the valve opening. However, at −1500 V (Figure 4e1–e3), although there was almost no oxide remaining in the channel, many bubbles appeared in the liquid metal channel because the higher voltage led to obvious water electrolysis (Movie S1), which also affected the normal transportation of the fluid. Therefore, −800 V is the adopted cathodic protection voltage, considering repeatability of the microvalve switch and smooth transportation of fluid.

### 3.3. Repetitiveness

To verify the improvement of repeatability by cathodic protection, cycle switching tests were performed on the microvalves. The specific process was as follows: When the negative protection voltage remained at −800 V, the pressure of the liquid metal and Di water were set to 400 mbar and 100 mbar, respectively. At this moment, the valve started to close, and the waiting time was set to 20 s. Then, the liquid metal valve pressure was set to 100 mbar and the Di water pressure remained unchanged, so that the valve started to open, and the waiting time was set to another 20 s. This process acted as a cycle, during which the flow rate was continuously recorded. Thereafter, we repeated this cycle and continuously observed the valves’ opening and closing conditions by using a microscope until the valve failed to work, reflecting a significant change in flow rate. It is noted that the procedure described above is controlled by the programmable software “Microfluidics Automation Tool” connected to the four-channel microfluidic control system.

**Figure 5:**
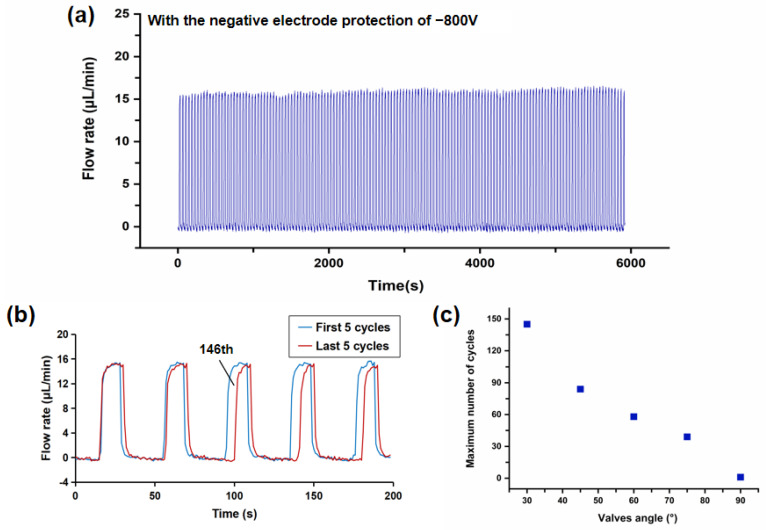
Repeatability test of microvalves. (**a**) Cycle switching tests for the valve and amplification curve for last 5 cycles. (**b**) The comparion of the first 5 cycles and the last 5 cycles. (**c**) Maximum number of cycles for different valve angles.

After several tests, the microvalve with the 30° angle had the best repeatability, and could complete up to 145 on/off switch cycles under −800 V; the result is shown in Figure 5a,b and Movie S2. In Figure 5a, each time the curve passes one peak represents one valve opening, and each time it passes one trough represents one valve closure. A total of 148 on/off switch cycles were carried out in this test. However, in Figure 5b, during the 146th valve opening process, the time for valve opening is significantly delayed compared with previous cycles due to the large accumulation of residual oxides on the wall, and the average flow rate starts to decrease. Similar results are presented in the 147th and 148^th^ openings. This indicates that the valve opening function is obstructed after the 145th. Therefore, the maximum number of cycles is 145. For comparison, the maximum number of cycles for other microvalves at a voltage of −800 V in multiple tests could be seen from Figure 5c. It was 84, 58, 39, and 1, corresponding to 45°, 60°, 75°, and 90°, respectively (Movie S3–S6). The microvalve with the 90° angle was almost non-repeatable. This is because the larger the angle, the smaller the pressure applied on the liquid metal along the microchannel, thus the liquid metal has more difficulty in flowing back. For a 90° valve, the pushing direction is completely perpendicular to the direction of movement of the liquid metal, so it can hardly be fully opened.

### 3.4. Valve Tightness

**Figure 6:**
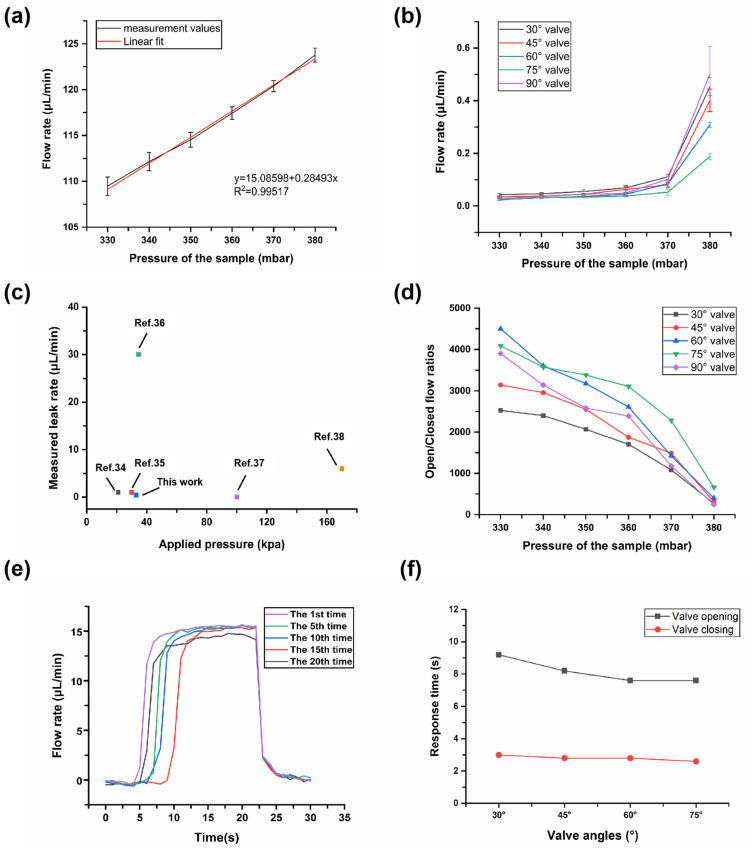
Leakage and response time tests of microvalves. (**a**) A line graph of sample flow rate as a function of pressure when the valve was opened. (**b**) A line graph of sample flow rate as a function of pressure when the valve was closed. (**c**) Comparison of our microvalve and other microvalves reported in the current literature on leak rate. (**d**) A line graph of the open/closed flow ratios of the valves under different pressures. (**e**) The relationship curve between the flow rate and time for one complete switching process of a 30° valve. (**f**) Response times for all valves of different angles.

Valve tightness is an indicator of valve performance, and is reflected in the leak rate and open/closed flow ratio. Leak rate is the flow rate when the valve is closed, and the open/closed flow ratio is acquired by Q_open_/Q_close_ (Q is the volume flow rate) when the sample pressure is constant. Figure 6a shows that the flow rate of the sample increased nearly linearly (R^2^ = 0.99517) as the sample pressure increased when the valve was opened. At a minimum pressure of 330 mbar, the flow rate was a minimum of 109.5 ± 1.0 μL min^−1^. When the pressure reached 380 mbar, the maximum flow rate was 123.8 ± 0.8 μL min^−1^.

After the valve was closed (400 mbar of the liquid metal), the data regarding the average flow rate of the sample at different pressures (one measurement point per 10 mbar) was recorded. At ≤320 mbar, no leak rate was detected by the flow rate platform, which proved excellent tightness of the valve. When the sample pressure reached 330 mbar, leakage occurred. Figure 6b shows the leak rates of the valves with different angles from 330 mbar to 380 mbar. When the sample pressure is 330 mbar, the average leak rate of each valve (from 30° valve to 90° valve) was only 0.043 ± 0.005 μL min^−1^, 0.035 ± 0.001 μL min^−1^, 0.024 ± 0.003 μL min^−1^, 0.027 ± 0.003 μL min^−1^, and 0.028 ± 0.004 μL min^−1^, respectively. In general, the leak rate of all the valves was ≤0.043 μL min^−1^ at 330 mbar. When the sample pressure changed from 370 mbar to 380 mbar, the average leak rates of all valves increased substantially and reached their maximum values, which were 0.454 ± 0.03 μL min^−1^, 0.401 ± 0.04 μL min^−1^, 0.309 ± 0.01 μL min^−1^, 0.188 ± 0.01 μL min^−1^, and 0.502 ± 0.10 μL min^−1^, respectively. This indicated that the valve tightness for each angle at 380 mbar became weak. In Figure 6c, we compared the leak rate of our microvalve (0.043 μL min^−1^ at 330 mbar) with others reported in the current literature [34,35,36,37,38]. It proves this work presents excellent anti-leakage characteristics. Figure 6d also shows the results of average open/closed flow ratios of all types of valves at different pressures. It could be seen that as the sample pressure increased, the open/closed flow ratio decreased. When the sample pressure was 330 mbar, the open/closed flow ratios were high (2527, 3144, 4501, 4087, and 3900, respectively, from 30° valve to 90° valve). Among them, the 60° and 75° valves had higher open/closed flow ratios. Even when the pressure reached 370 mbar, the open/closed flow ratio of each valve was above 10^3^, and this order of magnitude is sufficient for our microvalve to do some applications, such as bubble flow control. However, when the sample pressure reached 380 mbar, a substantial increase in leakage caused the open/closed flow ratios to become 273, 309, 401, 659, and 246, respectively. Therefore, to ensure the tightness of the valve, the sample pressure should be kept below 370 mbar.

### 3.5. Valve Response Time

Response time of the microvalve is reflected in the time required for the valve to open or close at one time. The test method was as follows: the microvalve executed multiple switch commands under the optimal cathodic protection of −800 V, while the relationship curve between the flow rate and time was recorded during the operation of open and close for the 1st time, the 5th time, the 10th time, the 15th time, and the 20th time. The time required for the flow rate to change from start to steady during the opening or closing process was regarded as the valve response time. The response time results of a 30° valve can be seen in Figure 6d. The response time results of all other angles, except for 90° (the 90° valve cannot be opened) are shown in Figure S2. The average response time required for the 30° valve opening was 9.2 s, and the average time required for the 30° valve closing was only 3.0 s. Figure 6e also shows the average response times of the valves with different angles. Among them, the 60° valve (7.6 s for opening, 2.8 s for closing) and 75° valve (7.6 s for opening, 2.6 s for closing) had faster response times than the others; this is because their liquid metal paths are shorter. Overall, the opening speed of the microvalve was slow, because the pressure value provided by our microfluidic control system dropped slowly, and it was also limited by oxide accumulation. However, sometimes a relatively slow response time increases the cushioning capacity of the microvalve, which effectively avoids water hammer caused by instantaneous pressure difference, thus preventing deformation or even rupture of the “liquid metal tongue” and improving the durability of the microvalve.

### 3.6. Burst Pressure

**Figure 7:**
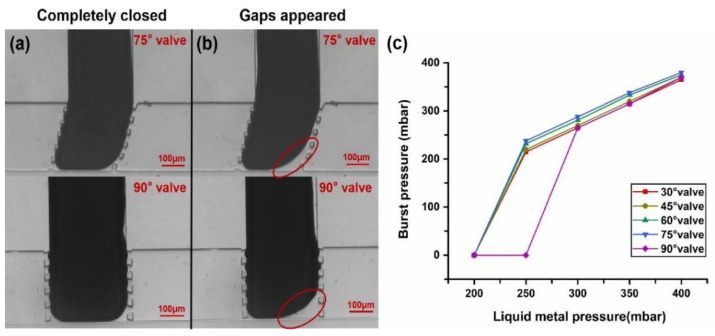
Morphological changes of microvalves at burst pressure and burst pressure values under different conditions. (**a**) The microvalves were completely closed. (**b**) Partial gaps appeared in the microvalves. (**c**) Burst pressure curve for the microvalves of different angles.

“Burst pressure” is the water pressure at which the deformation of the liquid metal tongue is larger than 10% when the valve is closed. It reflects the maximum pressure of the sample that the valve can withstand. When the water pressure was relatively low, the liquid metal could fill almost the entire vertical channel without voids (except some dead zones due to the contact angle) under certain liquid metal pressures, thus the microvalve was completely closed. Figure 7a shows the state of the microvalves when they were completely closed. When the water pressure gradually increased to a certain value, which was also the burst pressure value, it could be observed that the deformation of the liquid metal tongue was larger than 10%, as shown in the red circles of Figure 7b (Figure S3 and Movie S7 also show the phenomenon of burst pressure).

From Section 3.1, it is known that the proper pressure of the liquid metal for valve close is 400 mbar. Later, we also found that the liquid metal pressure to maintain the valve close is from 200 mbar to 400 mbar (except for the 90° valve, which is from 250 mbar to 400 mbar) if no water pressure is applied. Therefore, the burst pressure values corresponding to different liquid metal pressure values when the valve closes were studied (Figure S4 shows the state of a 30° microvalve at its different burst pressure values). The results from Figure 7c show that the burst pressures of all types of microvalves changed almost linearly with the liquid metal pressure values in a certain range (from 300 mbar to 400 mbar). The highest burst pressure was 385 mbar of the 75° valve when liquid metal was 400 mbar. However, when the liquid metal pressure was reduced to 250 mbar, the liquid metal tongue of the 90° microvalve started to flow back, so the valve could not maintain closure below 250 mbar even if no water pressure was applied; this means the burst pressure became 0. Other types of microvalves also began to flow back when the liquid metal pressure reduced to 200 mbar, so their burst pressures also become 0 below 200 mbar. This can be explained based on the high surface tension of liquid metal. When the pressure of the liquid metal is not high enough to overcome the surface tension, the liquid metal tongue will withdraw back to its microchannel even if the water pressure is 0 mbar. These results indicate that if we want the valve to withstand more pressure, a 75° valve with liquid metal pressure of 400 mbar is the best choice.

### 3.7. Application in Bubble Flow Control

**Figure 8:**
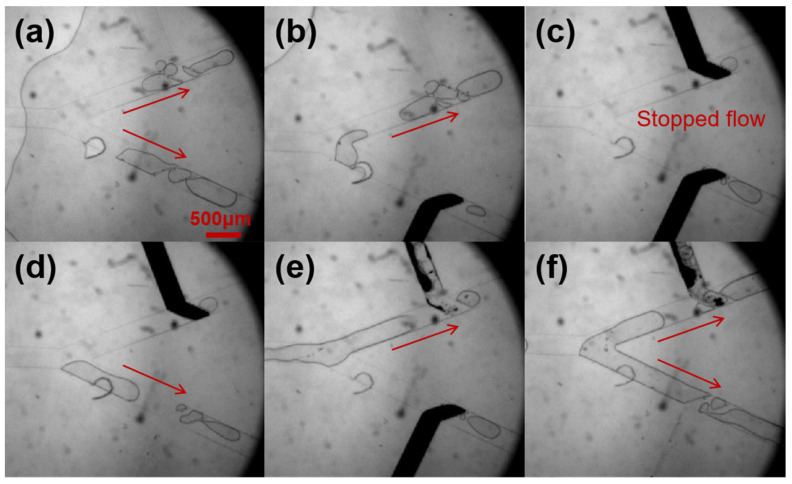
Bubbles’ flow direction manipulated by two microvalves in Y-shaped channel. (**a**) Before two valves were closed. (**b**) The lower side valve was closed. (**c**) Both the upper and lower side valve were closed. (**d**) The lower side valve was opened. (**e**) The upper side valve was opened. (**f**) Both the upper and lower side valve were opened.

Based on the study of liquid-metal-based microvalves, we also fabricated a chip that could control bubble motion. As shown in Figure 8, two liquid-metal-based microvalves were respectively integrated in the two branch channels of a Y-shaped channel. First, bubbles were generated by a T-shape channel. Di water worked as a continuous phase with pressure of 130 mbar; air worked as a dispersed phase with pressure of 160 mbar. Then the bubbles were delivered to the bifurcation of the Y-shape channel. When the bubbles reached the bifurcation point, the microvalve in the branch channel could control their flow direction (shown in Figure 8 and Movie S8).

Besides bubble flow control, we also believe that this microvalve can be used to control most water-based microflows. In addition, it can also be used for controlling the movement of droplet, particles, or cells in water-based microfluidic control. For flow control of biological samples, the applied voltage may cause damage; however, by designing the microchannel structure, for example increasing the distance between the microvalve and the sample area, the effect of voltage can be greatly weakened. Compared with other microvalves, this liquid-metal-based microvalve has a very simple structure where no multi-layer fabrication is needed, making this technology simple to implement. Besides, if the chip material is soft, this microvalve can also be totally soft. Because of its single-layer structure, this microvalve can be easily integrated into a very thin membrane, so it has advantages in stretching and bending, which is suitable for wearable devices in the future. Therefore, we believe this liquid-metal-based microvalve has a wide application prospect in microfluidic systems.

## 4. Conclusions

In this study, a room temperature liquid-metal-based microvalve with simple fabrication, easy operation, high flexibility, and a low leak rate was proposed and demonstrated. This microvalve can be designed and fabricated at the same time with a sample channel on the same chip layer, which overcomes the shortcomings of the traditional multi-layer microvalve structure that is normally complicated to fabricate. This microvalve can be closed or opened by controlling the pressure of the liquid metal according to the pressure of the sample. In order to eliminate the effect of metal oxide, which limited the lifespan of the microvalve, a cathodic protection method for liquid metal has been proposed and demonstrated which allows the microvalve to achieve as high as 145 cycles. In addition, this microvalve has a low leak rate that is ≤0.43 μL/min even if the fluid pressure is as high as 330 mbar, indicating that it has good open/close efficiency. Finally, the bubble flow control using this microvalve reflects its application prospect in the field of microfluidics.

## Supplementary Materials

The following are available online at https://www.mdpi.com/article/10.3390/mi12091051/s1, Figure S1: Principle of cathodic protection of the valve. Figure S2: The relationship curve between the flow rate and time for one complete switching process of other angle valves. Figure S3: All types of microvalves’ change under their burst pressures. Figure S4: Gaps occurred of 30° microvalves at different burst pressure values. Movie S1: Bubbles appeared in the microvalve at −1500 V. Movie S2–S6: Cycle switching test of microvalves with different angles at −800 V. Movie S7: Microvalve’s change under burst pressure. Movie S8: Application in bubble manipulation.

## References

[1] Glick C.C., Srimongkol M.T., Schwartz A.J., Zhuang W.S., Lin J.C., Warren R.H., Tekell D.R., Satamalee P.A., Lin L. (2016). Rapid assembly of multilayer microfluidic structures via 3D-printed transfer molding and bonding. Microsyst. Nanoeng..

[2] Grover W., Skelley A.M., Liu C.N., Lagally E.T., A Mathies R. (2003). Monolithic membrane valves and diaphragm pumps for practical large-scale integration into glass microfluidic devices. Sensors Actuators B Chem..

[3] Kim J., Stockton A.M., Jensen E.C., Mathies R.A. (2016). Pneumatically actuated microvalve circuits for programmable automation of chemical and biochemical analysis. Lab Chip.

[4] Li Z., He Q., Ma D., Chen H. (2010). On-Chip integrated multi-thermo-actuated microvalves of poly(N-isopropylacrylamide) for microflow injection analysis. Anal. Chim. Acta.

[5] Cooksey G.A., Sip C.G., Folch A. (2008). A multi-purpose microfluidic perfusion system with combinatorial choice of inputs, mixtures, gradient patterns, and flow rates. Lab Chip.

[6] Li N., Hsu C.-H., Folch A. (2005). Parallel mixing of photolithographically defined nanoliter volumes using elastomeric microvalve arrays. Electrophoresis.

[7] Zeng S., Li B., Su X., Qin J., Lin B. (2009). Microvalve-Actuated precise control of individual droplets in microfluidic devices. Lab Chip.

[8] Ottesen E.A., Hong J.W., Quake S.R., Leadbetter J.R. (2006). Microfluidic digital pcr enables multigene analysis of individual environmental bacteria. Science.

[9] Wang L., Li P.C., Yu H.-Z., Parameswaran A.M. (2008). Fungal pathogenic nucleic acid detection achieved with a microfluidic microarray device. Anal. Chim. Acta.

[10] Liu Y., Butler W.B., Pappas D. (2012). Spatially selective reagent delivery into cancer cells using a two-layer micro-fluidic culture system. Anal. Chim. Acta.

[11] Gomez-Sjoberg R., Leyrat A.A., Pirone D.M., Chen C., Quake S.R. (2007). Versatile, Fully Automated, Microfluidic Cell Culture System. Anal. Chem..

[12] Volpetti F., Garcia-Cordero J.L., Maerkl S.J. (2015). A Microfluidic Platform for High-Throughput Multiplexed Protein Quantitation. PLoS ONE.

[13] Anjewierden D., A Liddiard G., Gale B. (2012). An electrostatic microvalve for pneumatic control of microfluidic systems. J. Micromech. Microeng..

[14] Yoshida K., Tanaka S., Hagihara Y., Tomonari S., Esashi M. (2010). Normally closed electrostatic microvalve with pressure balance mechanism for portable fuel cell application. Sens. Actuators A Phys..

[15] Li M., Li D. (2018). Microvalve using electrokinetic motion of electrically induced Janus droplet. Anal. Chim. Acta.

[16] Wu X., Kim S.-H., Ji C.-H., Allen M.G. (2011). A solid hydraulically amplified piezoelectric microvalve. J. Micromech. Microeng..

[17] Bozhi Y., Qiao L. (2009). A latchable phase-change microvalve with integrated heaters. J. Microelectromech. Syst..

[18] Luharuka R., LeBlanc S., Bintoro J.S., Berthelot Y.H., Hesketh P.J. (2008). Simulated and experimental dynamic response characterization of an electromagnetic microvalve. Sens. Actuators A Phys..

[19] Kohl M., Dittmann D., Quandt E., Winzek B., Miyazaki S., Allen D. (1999). Shape memory microvalves based on thin films or rolled sheets. Mater. Sci. Eng. A.

[20] Unger M.A., Chou H.-P., Thorsen T., Scherer A., Quake S.R. (2000). Monolithic Microfabricated Valves and Pumps by Multilayer Soft Lithography. Science.

[21] Gui L., Ren C.L. (2011). Exploration and evaluation of embedded shape memory alloy (sma) microvalves for high aspect ratio microchannels. Sens. Actuators A Phys..

[22] Lee Y.-S., Bhattacharjee N., Folch A. (2018). 3D-Printed Quake-style microvalves and micropumps. Lab Chip.

[23] So J.-H., Dickey M. (2011). Inherently aligned microfluidic electrodes composed of liquid metal. Lab Chip.

[24] Gao M., Gui L. (2014). A handy liquid metal based electroosmotic flow pump. Lab Chip.

[25] Li G., Lee D.-W. (2017). An advanced selective liquid-metal plating technique for stretchable biosensor applications. Lab Chip.

[26] Varga M., Ladd C., Ma S., Holbery J., Tröster G. (2017). On-Skin liquid metal inertial sensor. Lab Chip.

[27] Wang M., Trlica C., Khan M.R., Dickey M.D., Adams J.J. (2015). A reconfigurable liquid metal antenna driven by electrochemically controlled capillarity. J. Appl. Phys..

[28] Gao M., Gui L. (2016). Development of a fast thermal response microfluidic system using liquid metal. J. Micromech. Microeng..

[29] Debray A., Shibata M., Fujita H. (2007). A low melting point alloy as a functional material for a one-shot micro-valve. J. Micromech. Microeng..

[30] Shaikh K.A., Shifeng L., Chang L. (2008). Development of a latchable microvalve employing a low-melting-temperature metal alloy. J. Microelectromech. Syst..

[31] Pekas N., Zhang Q., Juncker D. (2012). Electrostatic actuator with liquid metal–elastomer compliant electrodes used for on-chip microvalving. J. Micromech. Microeng..

[32] Tang S.-Y., Lin Y., Joshipura I.D., Khoshmanesh K., Dickey M.D. (2015). Steering liquid metal flow in microchannels using low voltages. Lab Chip.

[33] Zhao X., Xu S., Liu J. (2017). Surface tension of liquid metal: Role, mechanism and application. Front. Energy.

[34] Oh K.W., Rong R., Ahn C.H. (2001). In-Line micro ball valve through polymer tubing. Micro Total Analysis Systems.

[35] Takao H., Miyamura K., Ebi H., Ashiki M., Sawada K., Ishida M. (2005). A MEMS microvalve with PDMS diaphragm and two-chamber configuration of thermo-pneumatic actuator for integrated blood test system on silicon. Sens. Actuators A Phys..

[36] Jerman H. (1994). Electrically activated normally closed diaphragm valves. J. Micromech. Microeng..

[37] Rogge T., Rummler Z., Schomburg W.K. (2004). Polymer micro valve with a hydraulic piezo-drive fabricated by the AMANDA process. Sens. Actuators A Phys..

[38] Yang X., Holke A., Jacobson S.A., Lang J.H., Schmidt M.A., Umans S.D. (2004). An electrostatic, on/off microvalve designed for gas fuel delivery for the MIT microengine. J. Microelectromech. Syst..

