# Supplementary material for: A Novel On-Chip Liquid-Metal-Enabled Microvalve
Source: Micromachines (Basel). 2021 Aug 30;12(9):1051. doi: 10.3390/mi12091051 (PMC8467270; doi:10.3390/mi12091051)
Supplement: Supplementary file 1 [file micromachines-12-01051-s001.zip › micromachines-1308949-SI final/micromachines-1308949-SI for proof.pdf]

micromachines

Supplementary materials

A Novel on-Chip Liquid-Metal-Enabled Microvalve

Jiahao Gong, Qifu Wang , Bingxin Liu , Huimin Zhang and Lin Gui

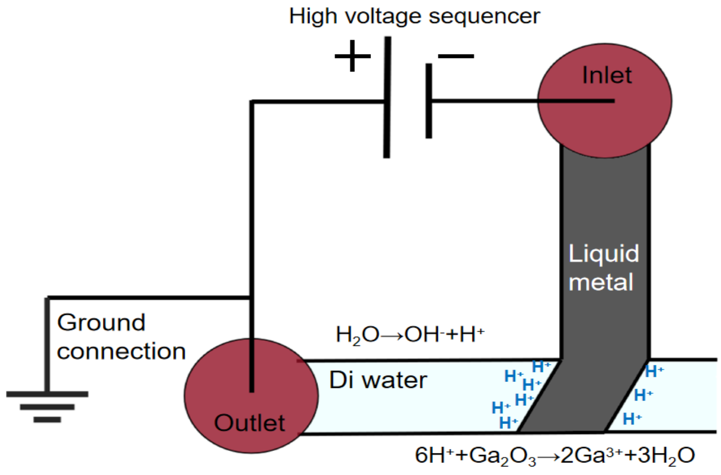

Figure S1. Principle of cathodic protection of the valve: A miniature electrolytic cell is constituted by liquid metal cathode, copper wire anode, and DI water. The oxide layer removal process is followed by: $H_2O \rightarrow OH^- + H^+$, $6H^+ + Ga_2O_3 \rightarrow 2Ga^{3+} + 3H_2O$.

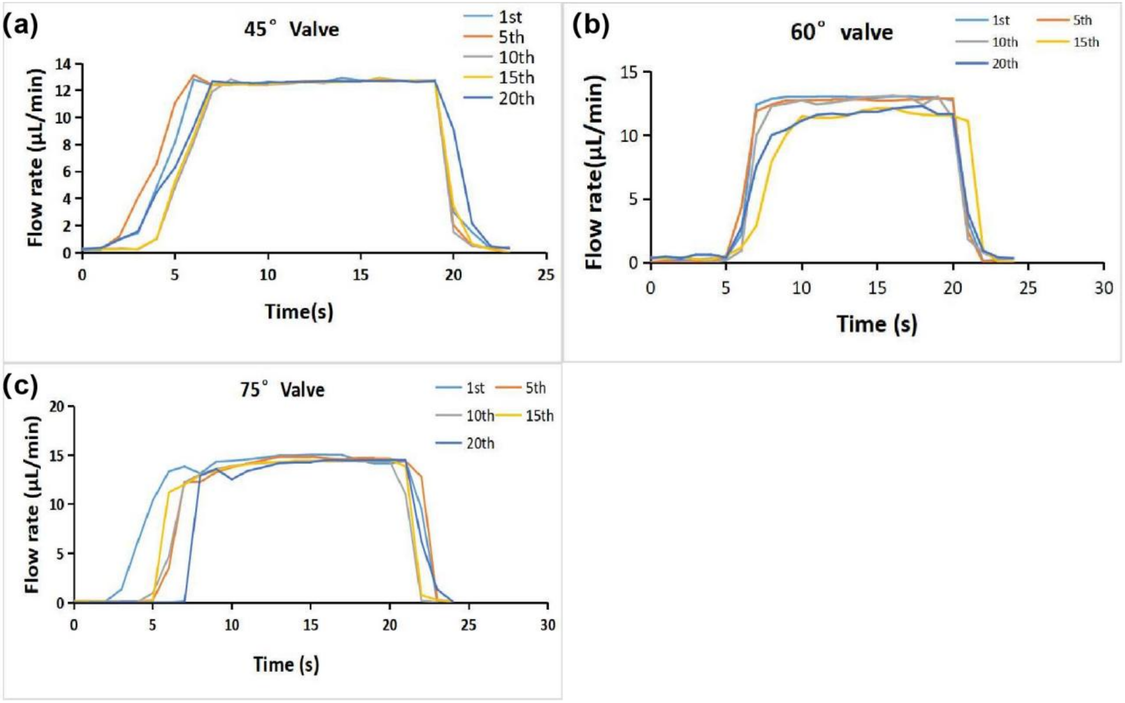

Figure S2. The relationship curve between the flow rate and time for one complete switching process of the valve with different angles.

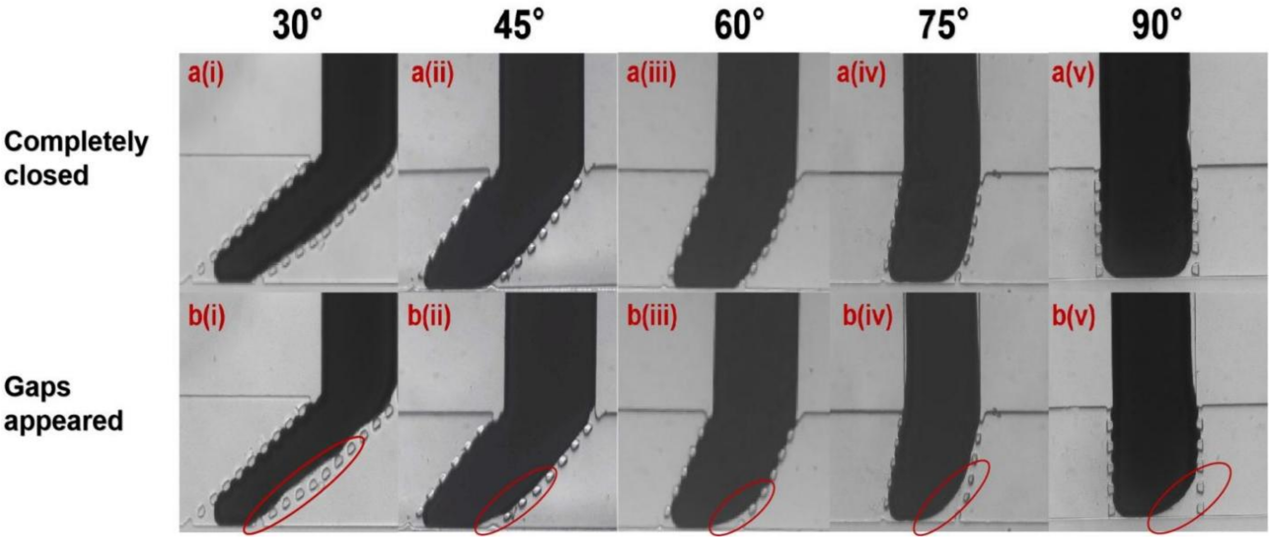

Figure S3. All types of microvalves' change under their burst pressures.

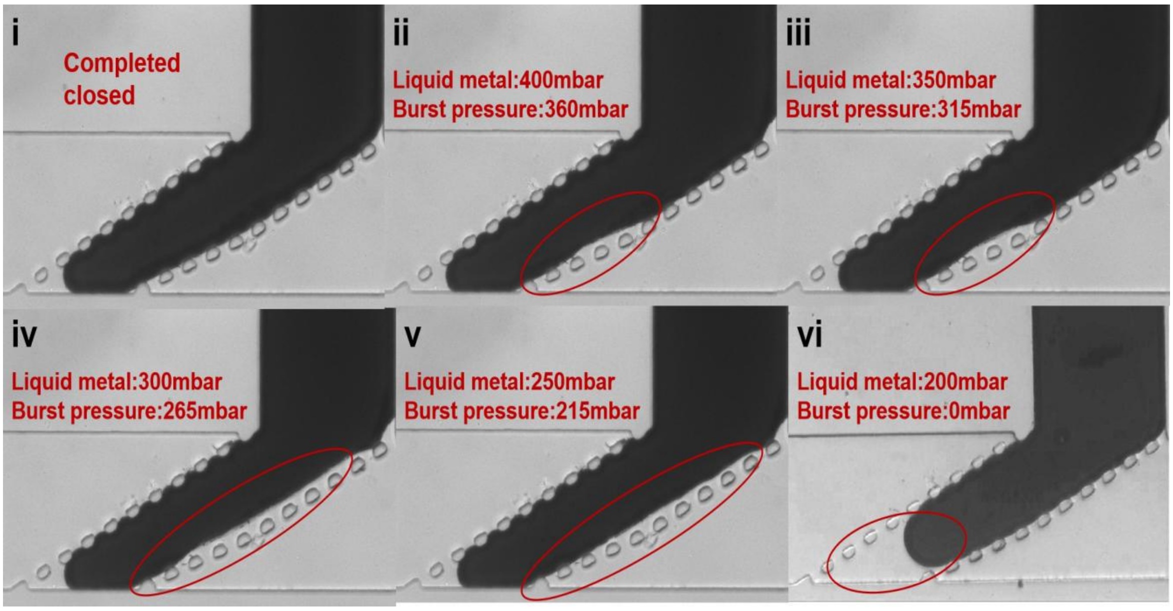

Figure S4. Gaps occurred of 30°microvalves at different burst pressure values. (i) the valve was completed closed at 400mbar. (ii-v) gaps occurred at different burst pressure values. (vi) the burst pressure became 0 when liquid metal pressure was set to 200mbar.
